# Studying Entrepreneurship-as-Practice Visually: Data Strategies and Analytical Considerations

**DOI:** 10.3389/fpsyg.2022.751270

**Published:** 2022-02-17

**Authors:** W. G. Will Zhao, Lina Ba

**Affiliations:** ^1^Faculty of Business Administration, Lakehead University, Thunder Bay, ON, Canada; ^2^Centre for Research in the Behavioural Sciences, Nottingham University Business School, Nottingham, United Kingdom; ^3^Institute for Organizational Research and Innovations (IORI), Wuhan Technology and Business University, Wuhan, China; ^4^School of Management, University of Bath, Bath, United Kingdom

**Keywords:** visual turn, practice turn, entrepreneurship-as-practice, data sources, data collection, analytical considerations

## Abstract

The objective of this essay is to forge a more explicit link between the “visual turn” and the “practice turn” in entrepreneurship research. Specifically, we explore three key aspects of mobilizing visual methods for studying entrepreneurship-as-practice (EaP), i.e., data sources, collection strategies, and analytical perspectives, highlighting the important theoretical and empirical promises that visual methods hold for said research. This essay bears implications for researchers and educators working at the intersection of entrepreneurship research, the practice theory, and visual methods.

## Introduction

Of late, the social science tradition of practice (Schatzki, 2001) has gained tremendous traction among entrepreneurship researchers for its potential to re-contextualize entrepreneurial practices and processes, and this ‘‘practice turn’’ has spawned a vibrant body of research^1^ collectively labeled as “Entrepreneurship-as-Practice (EaP)” (Claire et al., 2020; Thompson and Byrne, 2020; Thompson et al., 2020; Teague et al., 2021). Departing from conventional entrepreneurship research’s ontological individualism, i.e., defining entrepreneurial behavior as searching, perceiving, creating, or selecting opportunities, EaP research adopts a unique social ontology (Nicolini, 2017) where entrepreneurship-related social phenomena, including knowledge, meaning, action, etc., are understood as occurring within the nexus of the practices (Hui et al., 2016), and researchers preoccupy themselves with the day-to-day practices of entrepreneurs, focusing on the real-time unfolding and experiencing of said practices (Thompson et al., 2020).

Parallel to the “practice turn” was the “visual turn” that has been gaining momentum over the last 10 years in the broad field of management (Bell and Davison, 2013; Meyer et al., 2013). This intellectual movement represents researchers’ commitment to seriously engaging the visual-rich nature of our organized life and to extending the scope of data beyond the boundary of the commonly studied verbal texts. The growing prominence of the visual turn reflects the enhanced understanding of our field about the potency of visual artifacts in shaping, facilitating, and embodying our organizational life. Visuals, *static or not*, are no mere appendages of the verbal texts, but also are basic modes of social meaning construction, maintenance, and transformation. Many social science disciplines adjacent to entrepreneurship, e.g., strategic management (Knight et al., 2018) and organization studies (Höllerer et al., 2019), have taken up the challenge of incorporating the visual mode into empirical work. However, except for a handful of exceptions (Clarke, 2014; Ormiston and Thompson, 2021; Thompson and Illes, 2021), entrepreneurship research, even those inspired by the “practice turn,” has yet to engage visual methods seriously, despite the vast common ground in the analytical foci between these two “turns.”

It is, therefore, the objective of this essay to elaborate on some of the key empirical considerations when studying EaP with visual methods, and thereby to provide some clues for researchers interested in bringing together the visual turn and the practice turn in entrepreneurship research. Specifically, in the remainder of this article, we begin with an exposition of data sources, collection strategies and analytical considerations in visual research of EaP. We then illustrate our exposition with a vignette. We conclude this essay with its implications and a call for further visual research of EaP.

## Studying Entrepreneurship-As-Practice Visually: Data Sources, Collection Strategies, and Analytical Considerations

The practical approach treats our social world as being made up of interrelated and interdependent practices which become durable by being inscribed in the body and mind, objects and texts (Claire et al., 2020). Some existing entrepreneurship research has already started to connect the term “practice” to social ontology (Johannisson, 2011), which views the social as “a field of embodied, materially intertwined practices organized around a common practical understanding” (Schatzki et al., 2005:3).

Different entrepreneurs often replicate practices in specific environments at different times (Teague et al., 2021). It is these practices, rather than individuals, groups, institutions or networks (Nicolini, 2011), that constitute the unit of analysis in EaP research. This practice-oriented understanding of entrepreneurship challenges entrepreneurship researchers to extend their lens beyond the use of discourses and to include as many meaning-laden actions as possible into the scope of analysis (Clarke, 2014).

Visuality presents an important dimension of said meaning-laden actions. Entrepreneurship is a process full of visuals and visual practices. The day-to-day work of entrepreneurs in the process of starting and managing a business may be best captured in static and moving visuals. Equally important, a wide variety of visual artifacts (such as business-model canvas, product sketch) are produced in entrepreneurial processes. These visual data are rich and highly heterogeneous: some are processed by professional visual designers, some are images or footage taken by the researchers about the lives of entrepreneurs and their team members, and some others may be artistic expressions of the inner world of an entrepreneur. We argue that, by better understanding these different data sources, and their collection and analytical strategies, researchers can better understand the interrelated and interdependent practices inscribed in entrepreneurs’ bodies and minds, objects, and texts.

Much of the visual research tradition that we could readily mobilize in EaP research today originates from anthropology and documentary photography. Research has long argued that visual research excelled over other research methods in the way in which data was collected and used (Collier and Collier, 1986). Although it may appear abstract, photography can be an excellent supplement or even alternative to textual field notes. Meanwhile, the research situation can be much more faithfully restored in a video shot during the interview than could audio transcriptions. Beyond photography and video, visual data in social sciences can come in a variety of other forms, ranging from drawings, sketches, and other tangible visual artifacts to more abstract representations such as color or typography (Meyer et al., 2013). Such a diversity of visual data could provide much-needed empirical support as researchers seek to reconstruct the contexts for a startup or explain the motivations of an entrepreneur.

### Data Sources

Visual data could help researchers to reconstruct the real situation of entrepreneurship practices, and to generate insights that may not otherwise be available from analyzing verbal texts. Three categories of visual data are particularly important for EaP researchers to consider.

Category I: researcher-generated visual data. Here, the researcher’s primary visual task is not to collect and analyze visual artifacts as secondary data. In contrast, researchers have more active control over data generation. For each research situation, the researcher chooses the scene and records the interaction process in the form of static or moving visuals. One early research example of this kind was a 1998 study of a medical organization in the United Kingdom, where the researcher took 150 photographs of the interactions with patients as primary data (Buchanan, 1998). The images enabled the researcher to reconstruct the patients’ footprint, from referral, hospitalization, treatment to discharge. In a subsequent methodological paper, Buchanan (2001) elaborated on the visual approach used and noted that photographic records could afford researchers a deeper understanding of the lived experiences of informants.

Category II: participant-generated visual data. In more conventional visual research, this type of data is typically generated at the prompt of the researcher (Pauwels, 2010). For instance, research participants may be asked to create a visual with the verbal request, “Please draw a typical working day of your startup team.” The participants would then produce drawings accordingly. In EaP research, it is important to keep in mind that research participants also create visuals as part of their own entrepreneurial practices without the prompt of the researcher, as visual communication is the quasi-default mode of communication for entrepreneurs and teams (e.g., product sketches and value proposition canvases). Compared with the first category of data source, the researchers often have only limited control over data generation. Nevertheless, participants-generated data reveal the mindset of the participants in a more concrete manner. It is noteworthy that visual data can also lead to traditional text data, if the researcher reverses the above processes, i.e., presenting the participants with existing visual data, and asking them to respond to these visuals verbally (Wagner, 1979). For example, in a 1992 study on a hostile takeover, researchers used newspaper images to prompt participants to explain what they saw, and thereby obtaining psychoanalytic insights on the conflicts and uncertainties underlying the takeover (Schneider and Dunbar, 1992). Although this strategy is often referred to as “photo-elicitation,” researchers could use a variety of visual prompts, such as videos and artwork.

Category III: naturally occurring visual data. Such data had already been generated by third parties for other purposes than the research being conducted. The use of such data has a long history in management research. For example, in an early study of organizational beliefs, researchers analyzed customer photographs included in organizations’ annual reports to understand how organizations perceive their customers (Dougherty and Kunda, 1990). Another early study used a structured-symbolic cultural perspective to study meaningful actions, objects, and expressions in specific contexts, by analyzing public service advertisement images and the organization which produced them (Phillips and Brown, 1993).

### Collection Strategies

Like research in other management domains, the data collection strategies of EaP research are also largely dependent on the research objectives. At the empirical level, this means that researchers need to use different visual sampling and data acquisition strategies for different research questions. For data of Category I, visual research designs often begin with a “shooting script” (Suchar, 1992), which specifies the exact location, subject, and time. For example, a researcher conducting a diachronic study might record changes to the research object by retaking photographs with the same camera angle, camera distance, etc. (Rieger, 1996). Meanwhile, a hypothesis validation-driven research design might require a systematic, random, or stratified sampling strategy, such as periodically photographing the interior of a conference room (e.g., to capture pitching or networking practices). For the more exploratory research designs, a “grounded theory” approach may be more suitable (Collier and Collier, 1986), and researchers could take visual evidence (e.g., taking photographs) along the formation of a startup idea, the discovery of opportunities, etc.

Researchers need to be reminded that the practice approach is also process-focused, and therefore is best suited to decoding the entrepreneurial process (Johannisson, 2011; Watson, 2013), which is often more chaotic than linear, where initial ideas over time will be shaped by coincidences and the matters at hand through action and interactions (Baker and Nelson, 2005). Entrepreneurship is both full of moments of spark, but also of boredom.^2^ To capture and recognize the representative moment(s) requires EaP researchers to adopt a more “opportunity sampling” strategy (Sorenson and Jablonko, 1975), i.e., recording events of interest to the researchers when they occur or when they come into the researchers’ view. To better utilize this strategy, researchers also need to acquire as much contextual knowledge on the site that they study as possible (see more discussion in the subsection “Analytical Considerations”).

For data of Categories II and III, while researchers may be inclined to select specific data to collect based on their understanding of the research phenomenon, we argue that a more fruitful way may be to collect the data as they arise. Although developing a sensitivity to the empirical data is crucial, such sensitivity may be better exercised at the stage of data analysis rather than that of data collection. For example, when studying how startup teams evaluate different candidate business models, researchers should collect as many drafts and supporting visuals as possible, in addition to the final version of the business model decided by the team. Since visual data related to entrepreneurial practices are produced almost at all times, and because the cost of capturing and storing visual data has been significantly lowered over the last decade, researchers can build massive datasets with little effort.

Another important consideration, which pertains to ethics, is the intrusiveness nature of data collection (especially for Categories I and II). Granted, researchers need to clearly inform the participants of the existence of visual capturing devices, the research use of the visuals, and the participants’ rights before the start of data collection, in addition to abiding by the normal research ethics protocol. Yet, as many scholars (e.g., Warren, 2002) point out, the very act of holding a camera straight to one’s eyes or pointing it at someone is an invasive act that is hard not to pay attention to by research participants. When studying EaP, access to entrepreneurial practices *via* visual means may be more difficult than data collection methods that rely on verbal means, although today’s participants are arguably more likely to tolerate being videotaped than ever before, as they have grown accustomed to, for example, amateur video shooting from ubiquitous mobile phones and handheld devices (Clarke, 2014).

Furthermore, the presence of recording devices may also be a threat to bystanders, who are not being studied, as images and videos can reveal sensitive aspects of individuals and organizations, such as the faces of people, the geographical location of enterprises, product prototypes, and logo design. The issue of anonymity and confidentiality caused by the instant recognition of visuals needs to be paid more attention to when visually studying EaP. Related to this, some elements captured inside a visual may be protected by copyright law. Therefore, researchers need to anonymize their data by blurring faces, distorting objects, omitting names and geographic details, *even as they collect these data*.^3^ In general, researchers could use digital image processing applications installed on the mobile phone or other smart recording devices to process the visuals for anonymity and confidentiality purposes.

### Analytical Considerations

The extant literature has established three prominent perspectives from which visual researchers could view their data (Preston et al., 1996). First, images and other visual artifacts reflect social reality. Second, visual artifacts can conceal or distort social reality. Third, images are seen as constituting social reality in and of themselves. It is worth noting that most of the existing management studies take the first or the second perspective or their mixture; in other words, they study the relevance of visuals for understanding organization and management realities. For example, an early study explored how organizational members attempted to gain power and maintain control by advocating a specific dress code (Pratt and Rafaeli, 1997). The researchers found that the emphasis on the dress was a powerful symbol of organizational values, while the debate among small groups over dress codes is the symptom of deeper conflicts of social identities. In a study on the corporate social responsibility of Austrian public listed companies (PLCs), Hollerer et al. (2013) collected large-scale image samples and demonstrated how the corporate logic represented in these visual artifacts could be reproduced. As another example, Kaplan (2011) studies PowerPoint as a strategic cognitive mechanism, which is deeply implicated in the formation of organizational culture and is associated with group interests.

Unlike the individual interpretive studies of entrepreneurship which develop the understanding of entrepreneurial cognition and perception by prioritizing the analysis of narratives and discourses extracted from interviews and texts (Thompson and Illes, 2021), the practices that concern EaP scholars are, in Nicolini’s (2013) terminology, the discursive-material practices which bring meaning and intentionality into the action scene and which provide the participants with ways to influence each other and the social situation. To visually study networking, for example, means to derive the meaning of what we visually capture from networkers’ social situations in connection to other practices prior to and following a networking situation.

We argue that one of the advantages of studying EaP with visual methods lies precisely in that researchers are afforded an opportunity to capture not only the interaction observed, but also the context and process of phenomena unfolding. Yet, when analyzing the data collected, it is worth remembering that the visual data that the researchers collected (especially Category III) may not have been produced for the research projects. The researchers need to sensitize themselves about the specific context in which the data were generated, before embarking on data analysis. This is because, in the post-analysis of such visual data, they will rely on this contextual information to accurately evaluate the connotation and denotation of a visual artifact (Zhao, 2017). Just as visual art critics would do when analyzing “anonymous” visual art, EaP researchers often need to consult experts with relevant background knowledge, e.g., investors, employees, and entrepreneurs themselves, so as to gain deeper insights into entrepreneurs’ embodied experience with other social actors in the research site. In the following section, we briefly illustrate the key considerations that we have discussed so far with the example of a startup boot camp.

## Studying Entrepreneurship-As-Practice Visually: A Vignette

The startup boot camp, which forms the basis of our illustration, was offered in 2017 to startup entrepreneurs who were interested in enhancing their entrepreneurial skills with the Design Thinking approach. Participants of this 3-month camp reconvened 2 days each week to work on a wide spectrum of entrepreneurship-related tasks, including opportunity identification, team building, business model design, product prototyping, market analysis, resource identification and acquisition, pitching, and so on. From the beginning of the camp, the participants were made aware that their main objectives were to develop a business idea that would have market potential and to identify suitable organizational and financial strategies that could help exploit this idea.

**FIGURE 1:**
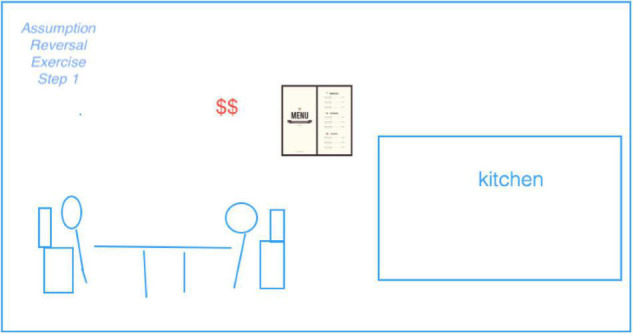
Some assumptions underlying the mainstream restaurant business model.

Our data collection was motivated by an initial, rather general research objective: How to interpret the taking shape of entrepreneurial ideas from the practice perspective. We conducted all the visual research activities mentioned above during the entirety of the boot camp, including documenting the day-to-day practices of entrepreneurs by taking photographs or videos, collecting relevant visual data as they arose or as they came into our attention (in addition, we also wrote conventional field notes). Particularly, when conducting interviews, we utilized both visual artifacts generated by us and those by the participants as prompts (i.e., photo-elicitation). During the same period, we collected a vast variety of visual artifacts produced by the entrepreneurs and their teams either by hand or by working together on a computer. Figure 1 is an example of such visuals, which was produced in the first phase of the ‘‘Assumption Reversal Exercise’’^4^ that entrepreneurs did to innovate the restaurant business model.

After each round of field observation and data collection, we would compile our field notes and review the data for the day before conducting preliminary labeling and grouping of the data. Adopting a visual lens on practices means that we would de-focus on the discourses and narratives but instead focus on how practices were *actually* created and reshaped through bodies, artifacts, texts, and their connections (Teague et al., 2020). Specific to our boot camp observations, it was clear to us that the submitted version of a business idea was shaped by a host of interconnected factors such as the business background of team members and their skills in identifying or framing opportunities, entrepreneurs’ ability to establish their authority, the materiality of the communication media used by the team, and the evolution of team dynamics. For example, we observed significant changes in team dynamics when brainstorming was conducted online, as compared to face-to-face, physical settings.

While many researchers may wish to skim through visuals as quickly as they can, visual analysis requires more careful reading, with proper attention to detail. This is because the meaning of visuals lies in not only what it reveals, but also what it implies^5^, i.e., the correct interpretation of a visual is achieved by being able to consciously read and respond (Becker, 1974). When analyzing visuals, the practice focus allows researchers to go beyond the content of the data they collect, more critically examining the context and reconstructing meaning on a more fundamental level, i.e., highlighting the deep structure of a group. For example, we could gain insights into the expressed and the implied meanings of Figure 1, thanks to our access to information on the actor (who contributed to the visual? was there anyone who dominated the creative process? Who talked more? Who drew/typed more?), the social relation (who were the participants?), the social situations (when and where was this visual generated?), symbol (what does the outer box mean?), and so forth.

Coffey (1999) argues that, for researchers that conduct fieldwork, “the self” is a pervasive constituent of their ethnographic inquiry. We believe this is especially true for visual researchers of EaP, who need to recognize that our presence and our bodies are also a central aspect of our activities in the research site. In addition to collecting and analyzing the various kinds of visual data obtained or generated during on-site interactions, we found it both natural and important to be reflective on our own identity and the social context of our research as we wrote our observation notes and analyzed our data. At times, it is necessary for such reflexivity to exercised collectively. For instance, when analyzing the same data, we needed constant discussions and comparisons of our interpretations, not only to achieve consistency of data interpretation, but also to allow a better understanding of our “selves” as researchers.

## Concluding Remarks

The objective of this essay was to make the connections of the visual turn and the practice turn more explicit in entrepreneurship research. It makes important contributions to our knowledge at the intersection of entrepreneurship research, the practice theory, and visual methods. On the one hand, by reviewing and outlining different types of visual data, this essay identifies the main empirical venues where EaP could be captured and subsequently analyzed. On the other hand, by discussing major analytical considerations and providing an illustrative vignette, this article constitutes one of our field’s early attempts to provide some empirical and methodological clues for studying EaP with visual methods.

Either as a source of data, or as a medium for the acquisition, processing and expression of social science knowledge, the visuals continue to challenge our field to reflect on how we can better capture, understand, and explain patterns of meaning construction, maintenance, and transformation (Jewitt et al., 2009). While qualitative research strategies such as non-participatory observation, shadowing, dialog analysis, and narrative analysis, are well suited to understanding the various day-to-day aspects of entrepreneurship (Claire et al., 2020), we argue that the practice approach requires researchers to carefully document and explain entrepreneurial process and practices in their social, historical, and cultural contexts, even as the phenomena develop in time and space, and to do so in a more self-reflective manner. For these reasons, this article calls for further visual research of EaP that more actively engages the visual turn so as to realize the great theoretical and empirical potential of the practice approach.

## Data Availability Statement

The original contributions presented in the study are included in the article/supplementary material, further inquiries can be directed to the corresponding author.

## Author Contributions

WZ: conception, review of literature, writing of the first draft, and revision. LB: review of literature and co-writing of the vignette section. Both authors approved the submitted version.

## Conflict of Interest

The authors declare that the research was conducted in the absence of any commercial or financial relationships that could be construed as a potential conflict of interest.

## Publisher’s Note

All claims expressed in this article are solely those of the authors and do not necessarily represent those of their affiliated organizations, or those of the publisher, the editors and the reviewers. Any product that may be evaluated in this article, or claim that may be made by its manufacturer, is not guaranteed or endorsed by the publisher.

## Footnotes

1 See Claire et al. (2020) for a systematic literature review of existing entrepreneurship-as-practice research.

2 We thank one reviewer for raising this excellent point.

3 Existing research has highlighted the ambiguity of the ownership of visual data generated during a visual research project (Pink, 2001). Instead of assuming that ownership is automatic because researchers have generated the visuals (e.g., taking the photographs), researchers need to clarify the ownership with participants and relevant organizations before including the visuals in research.

4 The Assumption Reversal Exercise is an ideation technique often used in entrepreneurship training, where new ideas are to be triggered from revising the existing assumptions ruling the current business solutions. Almost all business solutions (products, services, etc.) are designed and developed based on a set of assumptions. Breaking away from these assumptions would often lead to novel, creative solutions that are both profitable and sustainable. In this exercise, entrepreneurs were asked to perform three rounds of brainstorming and visualization around three questions: What are the prevalent assumptions of the current solution? What are some of the reversed assumptions? How do you make these reverse assumptions work in business?

5 We thank one reviewer for encouraging us to engage this point further.
